# Endoxifen for mammographic density reduction—results from the KARISMA endoxifen trial

**DOI:** 10.1093/jnci/djag087

**Published:** 2026-05-04

**Authors:** Per Hall, Mattias Hammarström, Jenny Bergqvist, Kamila Czene, Mikael Eriksson, Marike Gabrielson, José Tapia, Steven Quay, Stephen Nash, Magnus Bäcklund

**Affiliations:** Department of Medical Epidemiology and Biostatistics, Karolinska Institutet, Stockholm, Sweden; Department of Oncology, Södersjukhuset, Stockholm, Sweden; Department of Medical Epidemiology and Biostatistics, Karolinska Institutet, Stockholm, Sweden; Department of Medical Epidemiology and Biostatistics, Karolinska Institutet, Stockholm, Sweden; Department of Oncology, St Görans sjukhus, Stockholm, Sweden; Department of Medical Epidemiology and Biostatistics, Karolinska Institutet, Stockholm, Sweden; Department of Medical Epidemiology and Biostatistics, Karolinska Institutet, Stockholm, Sweden; Department of Medical Epidemiology and Biostatistics, Karolinska Institutet, Stockholm, Sweden; Department of Medical Epidemiology and Biostatistics, Karolinska Institutet, Stockholm, Sweden; Atossa Therapeutics, Seattle, WA, United States; Department of Medical Epidemiology and Biostatistics, Karolinska Institutet, Stockholm, Sweden; Department of Medical Epidemiology and Biostatistics, Karolinska Institutet, Stockholm, Sweden

## Abstract

**Background:**

(Z)-endoxifen is the tamoxifen metabolite that possesses the highest affinity to the estrogen receptor and is evolving as an alternative to tamoxifen. Mammographic breast density (MBD) change has been shown to be a proxy for tamoxifen therapy response. The objective was to measure the effect of 2 different doses of (Z)-endoxifen on MBD, safety, and side effects in healthy women.

**Methods:**

Healthy premenopausal women included in the national Swedish screening program in Stockholm were invited to KARISMA *Endoxifen*, a proof of principle, dose determining, double-blinded, randomized, placebo-controlled trial. Women were randomly assigned to placebo or 1 or 2 mg of (Z)-endoxifen daily for 6 months.

**Results:**

In all, 240 women were randomly assigned. There was a significant relative change in MBD in both (Z)-endoxifen arms compared to placebo: −19.3% (95% confidence interval [CI] = −6.15% to −32.4%) in the 1 mg arm and −26.5% (95% CI = −14.1% to −38.9%) in the 2 mg arm. The number of participants discontinuing because of adverse events related to the investigational medicinal product was 4 (placebo), 5 (1 mg), and 11 (2 mg), respectively. Participants on 2 mg of (Z)-endoxifen reported significantly higher scores of vasomotor symptoms, compared with placebo. No clinically significant changes in hematological safety tests or vital signs were noted.

**Conclusion:**

Both 1 and 2 mg of (Z)-endoxifen significantly reduced MBD to a degree comparable to the established 20 mg dose of tamoxifen. The 1 mg dosage of (Z)-endoxifen indicated superior tolerability. Future studies are necessary to confirm impact on breast cancer incidence.

**Trial Registration:**

ClinicalTrials.gov ID: NCT05068388.

## Background

Estrogens play a crucial role in regulating the growth and differentiation of glandular cells in the breast. Tamoxifen is a cornerstone in adjuvant treatment of breast cancer and exerts its effects by binding to the estrogen receptor, thereby blocking estrogen signaling.^1^ Although the Food and Drug Administration has approved tamoxifen for breast cancer prevention, its systemic side effects have limited its acceptance.^2^^,^^3^ Additionally, the therapeutic effect of tamoxifen is heterogeneous, because it acts as a pro-drug that must be metabolized in the liver.^4^ The most potent metabolite of tamoxifen, (Z)-endoxifen, has been proposed as an alternative treatment because it does not require activation and is less susceptible to drug interactions.^4^

Tamoxifen-induced reduction in mammographic breast density (MBD) is associated with therapeutic response and could serve as a measure of effectiveness.^5^^,^^6^ A decrease in MBD might also enhance the sensitivity of mammography, because high MBD can obscure a breast cancer.^7^

We conducted KARISMA *Endoxifen*, a proof-of-concept study, to test if (Z)-endoxifen had the same ability as tamoxifen to influence MBD area and to investigate safety and side effects. The trial was a double-blinded, randomized, placebo-controlled, 3-armed, dose determining, phase II trial of daily oral (Z)-endoxifen for 6 months. If (Z)-endoxifen proves to be as effective as tamoxifen while causing fewer side effects, it could enhance prevention efforts and increase the sensitivity of both 2D and 3D mammograms.

## Patients and methods

### Study design

KARISMA *Endoxifen*, was a proof-of-concept, dose determining, double-blinded, randomized, placebo-controlled, 3-armed, phase II trial of oral (Z)-endoxifen in premenopausal women. Participants were randomly assigned to daily capsules of placebo, 1 mg, or 2 mg (Z)-endoxifen for 6 months. This superiority study aimed at providing evidence that one or both active doses of (Z)-endoxifen were superior to placebo in reducing MBD area.

The trial was approved by the Swedish Ethical Review Authority (2022 to 02529-02) and Swedish Medical Products Agency (5.1 to 2021-29292).

### Participants and procedures

Women aged 40 to 55 years conducting a screening mammogram at 3 hospitals in Stockholm, Sweden, were invited. Recruitment began in December 2021 and ended in November 2023. Eligible participants were premenopausal women, defined as having at least 1 menstruation during the prior 12 months. If not conclusive, a blood-based FSH test was performed. A screening mammogram had to be performed within 3 months of inclusion and mammographic density assessed as above BI-RADS score A. Women of childbearing potential were urged to use an effective method of birth control throughout the study period and were willing to comply with monthly pregnancy testing. Further, women using agents that have the potential to decrease endoxifen levels through increased metabolic clearance were not eligible—for example, certain antiepileptic and antibiotic therapies.

A detailed description of eligibility criteria and the randomization procedure is available online (ClinicalTrials.gov: NCT05068388).^8^ All participants signed a written consent form before study entry.

Full-field digital mammograms of the mediolateral oblique view, from left and right breasts, were collected at baseline, 3 months, 6 months, or at early discontinuation. Mammographic breast density was assessed as area density (cm^2^) using the fully automated STRATUS method.^9^^,^^10^ Each participant’s MBD area of left and right breasts was measured and combined. Before MBD area measurements and comparisons were done, the images were aligned (positioned for optimal comparability) to reduce nonbiological variability as previously described.^11^

Safety was evaluated through vital signs and blood chemistry. Adverse event (AE) reporting was open for participants from study entry until 1 month after end of study medication, via a digital application, the KarmApp,^12^ phone, email, or visits at the study center. Tolerability was assessed through self-reported symptom scores using the 30-item questionnaire Breast Cancer Prevention Trial Eight Symptom Scale (BESS Plus 2007).^13^ We added 6 questions on tamoxifen-related symptoms previously identified in the KARISMA *Tamoxifen* trial^14^ (Table S1). A 5-graded Likert score was used (0 = “not at all,” 1 = “a little bit,” 2 = “somewhat,” 3 = “quite a bit,” and 4 = “very much”), and results at baseline were compared with results at the end of treatment. Missing answers in the questionnaire were excluded from the analyses.

### Randomization and masking

A total of 240 participants were block randomized (block size = 6) to a 1:1:1 ratio to receive placebo, 1 mg, or 2 mg of (Z)-endoxifen. All study participants, personnel, clinicians, and investigators were blinded to treatment allocation. When follow-up ended for the last participant, the database was locked and randomization information and plasma concentrations of (Z)-endoxifen added to the dataset.

### Statistical methods

This was a superiority study, which aimed to provide evidence that one or both of the active doses of (Z)-endoxifen were superior to placebo for reducing MBD area. The original total sample size estimation indicated 165 participants were required to provide 90% power with an alpha of .05 assuming an absolute effect size of 6.9 cm^2^ and standard deviation of 12 cm^2^, with the intention of using the Williams test to identify the minimum effective dose.^15^ This was inflated to 80 per arm to allow for dropouts, hence a total recruitment target of 240. Initially, *absolute* MBD change was selected as the primary endpoint. However, part way through the trial and before any outcome data had been assessed, it was decided to instead use *relative* MBD change because it reduces the influence of baseline MBD, and to use a gatekeeping approach for the statistical analysis as described in a Statistical Analysis Plan (SAP). The SAP was uploaded to ClinicalTrials.gov before unblinding of the data. These changes did not alter the fundamental framework of the study and were included in the updated protocol and analysis plan before data were unblinded.

The statistical power was also recalculated and found to be 86%, assuming the equivalent relative effect of 24.6 percentage points and shared standard deviation of 42 and allowing for the same dropout rate.

In the gatekeeping approach, we first compared the 0 mg with the 2 mg group; if and only if that showed evidence (*P* < .05) of efficacy did we compare 1 mg with placebo. Comparisons were made using separate linear regression models, with the relative reduction in MBD area as the outcome, an indicator of randomized arm (placebo or active) and adjusting for baseline MBD area. No other covariates were taken into consideration. As a secondary analysis we compared absolute change, using a linear regression model and adjusting for baseline MBD area. Safety data in terms of participant reported AEs were summarized using descriptive statistics. Tolerability was measured through change in Likert score from baseline to end of treatment, using linear regression with separate models for the 2 active doses and adjusting for baseline Likert score.

We performed analyses on both the modified intention-to-treat population (ITT) and per protocol population (PP), but the primary analyses were done using the ITT population. The ITT population was equal to the number of randomly assigned participants. The modified ITT population was defined as women with an MBD measured at baseline and at end of treatment (75 women in each arm). The PP population was defined as women who received at least 80% of the study drug to which they were randomly assigned (measured through pill count), had no major protocol deviations, conducted a baseline mammogram, and had at least 1 post-baseline mammogram. Safety endpoints were reported on the safety population, which consisted of all women who received at least 1 dose of their randomized treatment. All analyses were prespecified in the SAP.

Following the graphical presentations we have used previously,^16^ we produced exploratory post hoc plots of (Z)-endoxifen concentration (measured at 6 months) against (i) relative change in MBD (the primary efficacy outcome) and (ii) change in vasomotor symptom score (a key tolerability outcome included in BESS). For each outcome, we fitted a cubic spline model with knots at 2, 4, 6, 8, and 10 ng/mL. Splines were used to avoid imposing a linear or log-linear form of the association.

All *P*-values are 2-sided, and confidence intervals (CI) are at the 95% level. Analyses were conducted in Stata version 18.0 and SPSS version 28.

## Results

In total, 126 670 women were invited to the trial, 410 (0.3%) were screened for eligibility, and 240 (0.2%) were randomly assigned (Figure 1). One hundred and seventy participants were screening failures or excluded for other reasons (Figure 1). The most common reasons for noneligibility were postmenopausal status (*n* = 50) or a BMI >30 (*n* = 28). In addition, 32 women were entered into the electronic case report form (eCRF) but consent was not recorded, and 31 women gave consent and were found eligible but withdrew their consent before random assignment. A total of 42 participants discontinued, and 26 of these women did not perform an exit examination, leaving 214 women in the PP population (Figure 1).

**Figure 1.**
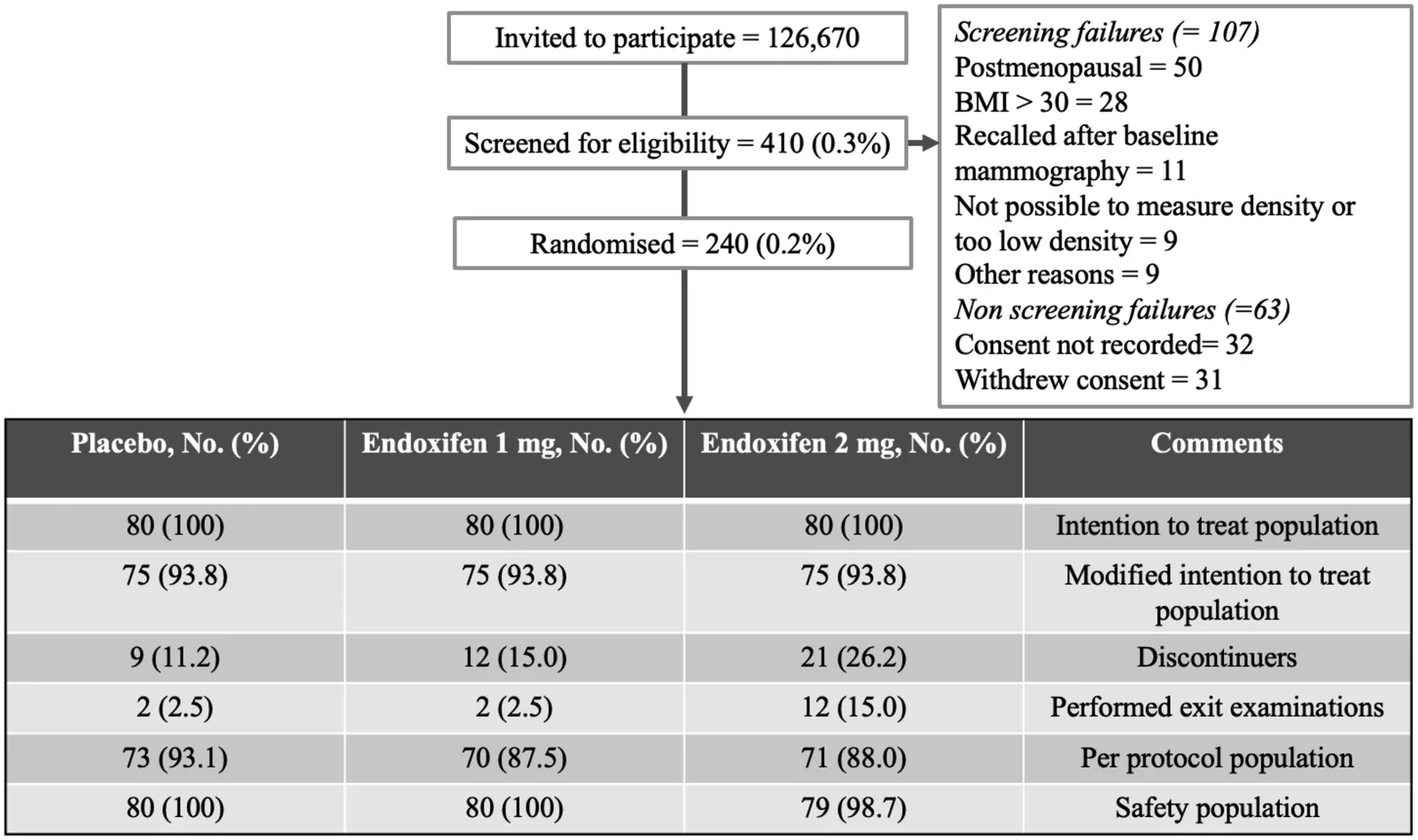
CONSORT diagram for the KARISMA *Endoxifen* trial.

No major differences in age at study entry, menarche, BMI, smoking prevalence, or family history of breast cancer were seen (Table 1). Additionally, no clinically significant changes in safety tests (serum chemistry, hematology, coagulation, urinalysis) or vital signs (physical examination including blood pressure and heart rate) were noted during the trial period (data not shown).

**Table 1.** Number of participants, age at study entry and menarche, BMI, smoking prevalence, family history of breast cancer, and vasomotor symptoms at baseline of the KARISMA *Endoxifen* trial stratified by randomization arms, in the intention-to-treat population.

		Randomization arms		
Baseline characteristics	All	Placebo	(Z)-endoxifen 1 mg	(Z)-endoxifen 2 mg
Number of participants	240	80	80	80
Age, mean (SD)	45.9 (3.6)	46.2 (3.5)	45.7 (3.7)	45.8 (3.5)
Age menarche, mean (SD)	12.8 (1.5)	12.8 (1.5)	12.9 (1.6)	12.9 (1.2)
BMI, mean (SD)	24.4 (2.9)	24.9 (3.0)	23.8 (2.8)	24.4 (2.6)
Regular smoking during the last year, number (%)	25 (10.4)	8 (10.0)	9 (11.2)	8 (10.0)
Breast cancer in family, number (%)	42 (17.5)	13 (16.3)	14 (17.5)	15 (18.8)
Baseline vasomotor symptoms, mean BESS score (SD)	0.43 (0.80)	0.44 (0.78)	0.33 (0.78)	0.52 (0.83)

Abbreviations: SD = standard deviation; BESS = Breast Cancer Prevention Trial (BCPT) Eight Symptom Scale.

The mean MBD area at baseline did not differ greatly between arms (43.8, 48.9, and 43.9 cm^2^ in the placebo, 1 mg, and 2 mg arms; Table 2). By the end of the trial, there was a substantial difference between the arms (44.7, 34.6, and 29.8 cm^2^, respectively). There was evidence of efficacy in the 2 mg group, with an estimated MBD decrease of −26.5% (95% CI = −38.8% to −14.1%; *P* < .001). As this cleared the prespecified gatekeeping approach, the 1 mg arm was analyzed. In the 1 mg group, the treatment effect was −19.3% (95% CI = −32.4% to −6.1%; *P* = .004; Table 2). Similar results were seen when analyses were restricted to the PP population (effect sizes were −28.8% and −18.2% in the 2 mg and 1 mg arms, respectively). When we compared absolute change in MBD, the results were a reduction of 5.9 cm^2^ (95% CI = −10.3 to −1.4) and 7.1 cm^2^ (95% CI = −11.0 to −3.2) in the 1 mg and 2 mg groups, respectively.

**Table 2.** Number of participants, baseline mean mammographic breast density (MBD) area and MBD area at end of treatment, percent change in MBD, and regression analyses in the modified intention-to-treat population.

	Randomization arms		
	Placebo	(Z)-endoxifen 1 mg	(Z)-endoxifen 2 mg
Number of participants (%)	75 (93.8)	75 (93.8)	75 (93.8)
Baseline MBD, mean area, cm^2^ (SD)	43.8 (26.5)	48.9 (31.3)	43.9 (25.1)
End of treatment MBD, mean area, cm^2^ (SD)	44.7 (29.9)	34.6 (22.4)	29.8 (18.9)
Percent change in MBD, mean (95% CI)	0.3 (−10.4 to 11.0)	−17.3 (−25.5 to −9.1)	−23.5 (−30.0 to −17.1)
Treatment effect (95% CI), *P* value	Ref.	−19.3 (−32.4 to −6.1), *P* = .004*	−26.5 (−38.8 to −14.1), *P* = <.001*

* Regression analyses were done in a 2-model approach comparing active dose with placebo and adjusted for baseline density.

Abbreviations: SD = standard deviation; CI = confidence interval.

The mean (Z)-endoxifen level at 6 months was 4.75 ng/mL in the 1 mg group and 9.69 ng/mL in the 2 mg arm (Table S2). There was large variation in both measured levels of (Z)-endoxifen in plasma and the response to treatment (Figure S1). Using a spline model, the mean relative MBD change reached a 20% decrease at (Z)-endoxifen of 3 to 4 ng/mL, with no substantial further decrease in MBD seen at higher concentrations (Figure 2). In a corresponding spline model, showing change in vasomotor symptom score (BESS), as a function of (Z)-endoxifen concentrations, there seemed to be an effect on symptoms at (Z)-endoxifen concentrations of 2 to 4 ng/mL. At concentrations higher than 8 ng/mL, there was an indication of a positive linear dose-response relationship between concentration and reported symptoms (Figure S2).

**Figure 2.**
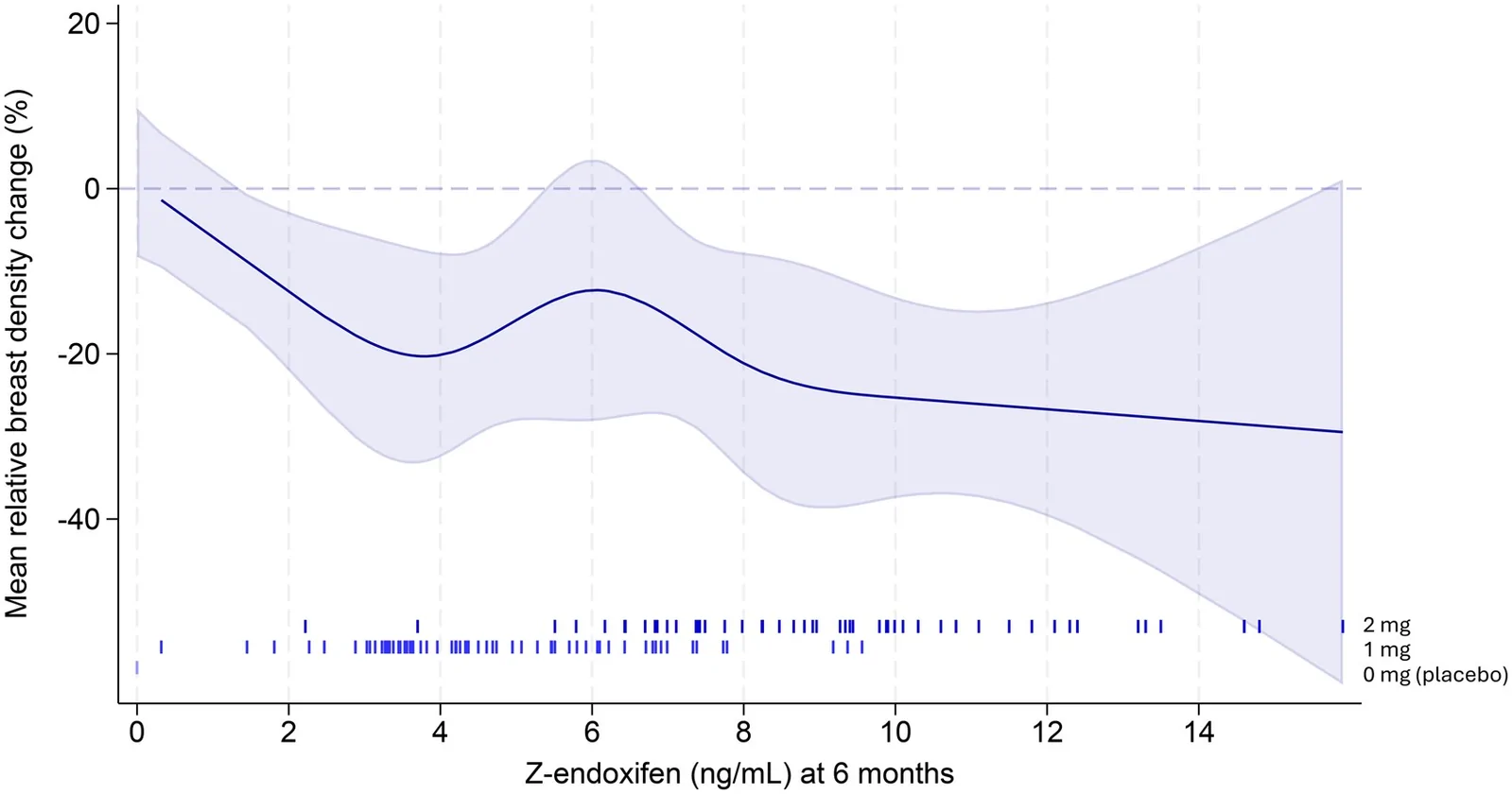
Spline model describing relative density change and endoxifen concentrations (ng/mL). (Z)-endoxifen doses in blue vertical dashes.

Mean days in study did not differ substantially between the 1 mg (189.1 days) and 2 mg (181.8 days) (Z)-endoxifen arms (Table 3). The total number of participants reporting an AE did not differ between randomization arms (Table 3). However, there were more participants reporting AEs related to the investigational medicinal product (IMP), as determined by the investigators, in the active arms: 32 and 34 in the 1 mg and 2 mg arms, compared with 16 participants in the placebo arm (Table 3). The most common AEs and, at the same time, the AEs with the largest differences between the active arms and placebo were irregular menstruation, night sweats, and hot flashes (Table S3). In all, 3 serious AEs (SAEs) were reported (Table 3): a breast cancer in the placebo arm, a subarachnoid hemorrhage in the 1 mg arm, and a myocardial infarction in the 2 mg (Z)-endoxifen arm. None of these SAEs were considered to be related to the IMP.

**Table 3.** Number of participants, mean days in study, number of participants reporting serious and non-serious adverse events (AE), related to investigational medicinal product (IMP), number of participants that discontinued and reason, stratified by randomization arm, in the intention-to-treat population.

	Randomization arms			
	All	Placebo	(Z)-endoxifen 1 mg	(Z)-endoxifen 2 mg
Number of participants	240	80	80	80
Mean days in study	186.1	197.2	189.1	181.8
**Adverse Events**				
Number of participants reporting AE (%)	205 (85.8)	66 (82.5)	69 (87.3)	70 (87.5)
Number of participants reporting AE related to IMP (%)	82 (34.3)	16 (20.0)	32 (40.0)	34 (43.0)
Number of participants reporting SAE (%)	3 (1.25)	1 (1.25)	1 (1.25)	1 (1.25)
**Discontinuation**				
Number of participants who discontinued (%)	42 (17.5)	9 (11.3)	12 (15.0)	21 (26.3)
Number of participants who discontinued due to an AE related to the IMP (%)	20 (8.3)	4 (5.0)	5 (6.3)	11 (13.8)

The total number who discontinued treatment was 42, and those who discontinued because of an AE considered to be related to the IMP was 4, 5, and 11, respectively (Table 3). The most reported AEs in this latter group of 20 participants were headache, night sweats, hot flashes, and feeling bloated, findings confined to women in the 2 mg arm (Table S4).

Using the validated BESS Plus questionnaire, including 8 symptom groups, and comparing baseline to end of treatment, we found a significantly increased mean score in vasomotor symptoms in the 2 mg arm (Table 4). When analyzing the full BESS Plus and KARISMA *Tamoxifen* questionnaire, item by item, we found that the difference in vasomotor symptoms was caused mainly by an increase in severity of reported night sweats and hot flashes (Table 4). Replies to the questionnaires indicated that participants randomly assigned to 1 mg of (Z)-endoxifen reported no worsening symptoms, but fewer problems from diarrhea, vaginal bleeding or spotting, blind spots, fuzzy vision, breast sensitivity/tenderness, dry mouth, and weight loss, when compared with placebo (Table 4). Participants randomly assigned to 2 mg reported fewer problems from joint pain and muscle stiffness.

**Table 4.** Number of participants, difference in mean score of reported symptoms, comparing baseline with end of treatment (at 6 months or at discontinuation) using the Breast Cancer Prevention Trial (BCPT) Eight Symptom Scale (BESS), BESS Plus, and KARISMA *Tamoxifen* questionnaires, and regression analyses of the difference between the active dose and placebo adjusted for baseline score, in the modified intention-to-treat population. Higher score means worse symptoms.

	*Change in symptom mean score per dose arm*			*Comparing means, active dose vs placebo*			
	Placebo	Endoxifen 1 mg	Endoxifen 2 mg	Endoxifen 1 mg vs Placebo		Endoxifen 2 mg vs Placebo	
Number of participants	80 (100%)	76 (95%)	77 (96%)				
**Symptom group**	**Mean (95% CI)**	**Mean (95% CI)**	**Mean (95% CI)**	**Effect**	***P***	**Effect**	***P***
**Cognitive (BESS)**	0.24 (−0.18 to 0.66)	−0.05 (−0.44 to 0.34)	0.32 (−0.16 to 0.81)	−0.243	.390	0.111	.723
*Subscale*							
Difficulty concentrating	0.09 (−0.09 to 0.26)	−0.03 (−0.20 to 0.14)	0.16 (−0.04 to 0.35)	−0.080	.482	0.098	.426
Easily distracted	−0.06 (−0.24 to 0.12)	−0.09 (−0.28 to 0.09)	0.05 (−0.14 to 0.25)	−0.018	.880	0.085	.486
Forgetfulness	0.21 (0.06 to 0.37)	0.07 (−0.08 to 0.21)	0.12 (−0.06 to 0.29)	−0.114	.259	−0.069	.533
**Muscular (BESS)**	0.13 (−0.33 to 0.58)	−0.30 (−0.75 to 0.14)	−0.30 (−0.64 to -0.05)	−0.361	.234	−0.451	0101
*Subscale*							
Joint pain	0.25 (0.03 to 0.47)	0.00 (−0.16 to 0.16)	−0.05 (−0.19 to 0.09)	−0.228	.086	−0.295	**.019**
Muscle stiffness	−0.01 (−0.20 to 0.18)	−0.24 (−0.46 to −0.02)	−0.23 (−0.42 to −0.05)	−0.169	.167	−0.246	**.034**
General aches and pains	−0.11 (−0.27 to 0.04)	−0.07 (−0.27 to 0.14)	−0.01 (−0.16 to 0.13)	0.056	.604	0.079	.404
**Vasomotor (BESS)**	0.24 (0.03 to 0.44)	0.64 (0.26 to 1.03)	1.14 (0.67 to 1.62)	0.351	.087	0.930	**<.001**
*Subscale*							
Night sweats	0.10 (−0.05 to 0.25)	0.26 (0.06 to 0.46)	0.58 (0.33 to 0.84)	0.134	.238	0.509	**<.001**
Hot flashes	0.10 (−0.02 to 0.22)	0.26 (0.10 to 0.43)	0.45 (0.24 to 0.67)	0.120	.203	0.350	**.004**
Cold sweats	0.04 (−0.01 to 0.08)	0.12 (0.02 to 0.22)	0.10 (0.00 to 0.21)	0.092	.071	0.081	.162
**Gastrointestinal (BESS)**	0.09 (−0.18 to 0.36)	−0.20 (−0.46 to 0.07)	0.03 (−0.16 to 0.21)	−0.335	**.007**	−0.219	.091
*Subscale*							
Vomiting	0.01 (−0.03 to 0.06)	−0.09 (−0.19 to 0.01)	−0.01 (−0.06 to 0.03)	−0.025	.174	−0.012	.594
Nausea	0.00(−0.14 to 0.14)	−0.03 (−0.14 to 0.09)	0.03 (−0.06 to 0.11)	−0.068	.336	−0.036	.612
Diarrhea	0.08 (−0.10 to 0.25)	−0.08 (−0.21 to 0.05)	0.01 (−0.12 to 0.14)	−0.222	**.009**	−0.146	.104
**Dyspareunia (BESS)**	0.15 (0.00 to 0.30)	0.29 (−0.03 to 0.61)	0.25 (0.05 to 0.44)	0.195	.202	0.115	.332
*Subscale*							
Vaginal dryness	0.11 (0.00 to 0.23)	0.24 (0.05 to 0.43)	0.21 (0.04 to 0.38)	0.164	.115	0.123	.220
Pain with intercourse	0.04 (−0.03 to 0.10)	0.05 (−0.11 to 0.21)	0.04 (−0.03 to 0.11)	0.019	.799	−0.005	.908
**Weight Concerns (BESS)**	−0.03 (−0.30 to 0.25)	−0.25 (−0.52 to 0.032)	0.06 (−0.23 to 0.36)	−0.160	.335	−0.74	.696
*Subscale*							
Weight gain	0.11 (−0.05 to 0.27)	0.12 (−0.04 to 0.27)	0.19 (0.01 to 0.38)	−0.017	.857	−0.022	.844
Unhappy with the appearance of my body	−0.14 (−0.32 to 0.05)	−0.37 (−0.56 to −0.18)	−0.13 (−0.30 to 0.04)	−0.152	.190	−0.073	.537
**Gynecological (BESS)**	0.04 (−0.33 to 0.40)	0.12 (−0.27 to 0.51)	0.10 (−0.25 to 0.46)	0.085	.733	0.084	.709
*Subscale*							
Vaginal discharge	−0.06 (−0.24 to 0.12)	0.05 (−0.16 to 0.27)	0.03 (−0.19 to 0.24)	0.140	.259	0.143	.229
Genital itching/irritation	0.05 (−0.11 to 0.21)	0.21 (0.01 to 0.41)	0.12 (−0.03 to 0.26)	0.168	.155	0.051	.599
Vaginal bleeding or spotting	0.05 (−0.13 to 0.23)	−0.14 (−0.31 to 0.03)	−0.04 (−0.19 to 0.11)	−0.230	**.023**	−0.105	.300
**Bladder Control (BESS)**	0.08 (−0.20 to 0.35)	0.03 (−0.22 to 0.27)	−0.23 (−0.49 to 0.02)	−0.110	.534	−0.261	.133
*Subscale*							
Difficulty with bladder control (when laughing or crying)	0.11 (−0.07 to 0.29)	−0.01 (−0.16 to 0.14)	−0.14 (−0.30 to 0.01)	−0.157	.156	−0.170	.124
Difficulty with bladder control (at other times)	−0.04 (−0.20 to 0.12)	0.04 (−0.12 to 0.20)	−0.09 (−0.26 to 0.07)	0.028	.779	−0.099	.317
**BESS Plus**							
Headaches	0.01 (−0.17 to 0.19)	−0.11 (−0.29 to 0.08)	0.00 (−0.21 to 0.21)	−0.151	.221	−0.044	.739
Blind spots, fuzzy vision	0.19 (0.06 to 0.32)	0.01 (−0.06 to 0.08)	0.10 (−0.02 to 0.22)	−0.209	**.002**	−0.128	.110
Breast sensitivity/tenderness	−0.07 (−0.26 to 0.11)	−0.18 (−0.32 to -0.05)	−0.35 (−0.53 to −0.17)	−0.185	**.042**	−0.181	.052
Weight loss	−0.05 (−0.20 to 0.10)	−0.08 (−0.18 to 0.02)	0.05 (−0.08 to 0.18)	−0.112	**.020**	−0.010	.880
Decreased appetite	−0.01 (−0.13 to 0.10)	0.08 (−0.02 to 0.18)	0.08 (−0.09 to 0.25)	0.030	.644	0.056	.473
Abdominal pain	−0.09 (−0.25 to 0.07)	0.13 (−0.07 to 0.34)	0.14 (0.02 to 0.27)	0.177	.082	0.137	.129
Pain or cramps in the legs or feet	0.14 (−0.01 to 0.29)	0.20 (0.04 to 0.36)	0.17 (0.03 to 0.31)	0.060	.579	0.022	.823
Back pain or problems	−0.01 (−0.17 to 0.15)	−0.05 (−0.26 to 0.15)	−0.03 (−0.16 to 0.11)	−0.103	.394	−0.071	.484
Low energy	0.15 (−0.04 to 0.34)	0.14 (−0.07 to 0.36)	0.27 (0.09 to 0.46)	−0.005	.970	0.069	.586
**KARISMA** *Tamoxifen*							
Rash	−0.10 (−0.22 to 0.02)	−0.05 (−0.16 to 0.06)	−0.06 (−0.21 to 0.08)	0.001	.986	−0.024	.709
Itching	−0.04 (−0.16 to 0.08)	0.08 (−0.06 to 0.22)	−0.13 (−0.25 to −0.01)	0.088	.244	−0.067	.242
Dry mouth	0.21 (0.11 to 0.32)	0.03 (−0.11 to 0.17)	0.13 (0.03 to 0.23)	−0.177	**.039**	−0.072	.324
Fatigue	−0.06 (−0.25 to 0.12)	0.01 (−0.22 to 0.24)	0.16 (−0.07 to 0.39)	0.046	.734	0.184	.192
Depression	0.20 (0.01 to 0.39)	0.11 (−0.08 to 0.29)	0.13 (−0.02 to 0.28)	−0.049	.683	−0.031	.539
Lost interest in sex	0.03 (−0.18 to 0.23)	0.01 (−0.26 to 0.28)	0.10 (−0.12 to 0.33)	−0.069	.624	0.085	.673

## Discussion

We showed that (Z)-endoxifen at doses of 1 and 2 mg, in contrast to placebo, significantly reduced MBD area. Approximately twice as many participants in the 1 and 2 mg (Z)-endoxifen groups reported AEs related to the IMP compared with the placebo group. However, the AEs did not seem to affect adherence in the lower dose since a similar number of women reported AEs leading to discontinuation in the placebo (*n* = 4) and the 1 mg (Z)-endoxifen (*n* = 5) groups. No significant difference between placebo and the 1 mg (Z)-endoxifen group in scoring worsening severity of 36 included symptoms of the BESS Plus and KARISMA *Tamoxifen* questionnaire was seen. In contrast, the 2 mg (Z)-endoxifen group reported significantly more night sweats and hot flashes compared with placebo.

In our previous KARISMA *Tamoxifen* trial,^14^ the same technique for measuring MBD was used. A similar reduction in MBD area, comparing 1 mg (19.3%) and 2 mg (26.5%) of (Z)-endoxifen to 20 mg (18.5%) of tamoxifen was seen, indicating that 1 or 2 mg of (Z)-endoxifen might be as effective as tamoxifen. A density decrease is associated with better prognosis among patients^6^ and a breast cancer preventive effect in healthy women.^5^

Comparing (Z)-endoxifen plasma concentrations from the current study with those observed in premenopausal women in the KARISMA *Tamoxifen* trial,^14^ participants randomly assigned to 1 mg of (Z)-endoxifen had a mean plasma concentration of 4.75 ng/mL (SD 1.89), similar to the 4.45 ng/mL (SD 2.63) observed in women who received 10 mg of tamoxifen, and above the mean concentration of 2.45 ng/mL (SD 1.12) seen in 5 mg tamoxifen (Table S5). For participants receiving 2 mg of (Z)-endoxifen, the mean concentration was 9.69 ng/mL (SD 2.84), compared with 11.20 ng/mL (SD 6.42) in those administered 20 mg of tamoxifen. The (Z)-endoxifen concentrations resulting from direct (Z)-endoxifen administration fell between the levels observed in intermediate and normal CYP2D6 metabolizers treated with tamoxifen.^16^

The variability in plasma concentrations was greater among those receiving tamoxifen^16^ compared with those on (Z)-endoxifen, as reflected by the larger standard deviations. As seen in Figure S1, the variation in (Z)-endoxifen concentrations, not explained by polymorphism of CYP2D6, needs to be explored further in future trials.

In a recent article, we identified a window of effect on mammographic breast density, defined as mean relative MBD decrease of −18.5%, seen in women randomly assigned to 20 mg of tamoxifen, at (Z)-endoxifen concentrations from 2 ng/mL with a nadir at ≈3 ng/mL.^16^ Reassuringly, our previous results were validated in the present study (Figure 2).

In the KARISMA *Tamoxifen* trial,^14^ we showed that 10 mg, 5 mg, and 2.5 mg tamoxifen decreased MBD in premenopausal women to the same extent as the 20 mg dose.^14^ In addition, the TAM-01 trial, including both pre- and postmenopausal women, showed that a tamoxifen dose of 5 mg had a preventive effect, but results were confined to postmenopausal women when stratifying for menopausal status.^17^  Figure S2 indicates lower symptom burden by lower (Z)-endoxifen concentrations. This favors the 1 mg dose in future studies.

The proportion of women reporting AEs was similar in the 3 treatment groups, but the number of women reporting AEs related to the IMP was twice as many in the active groups compared with placebo. The most commonly reported AEs in the (Z)-endoxifen groups were irregular menstruations, night sweats, and hot flashes. The total number of women discontinuing was 42: 9, 12, and 21 in the placebo, 1 mg, and 2 mg (Z)-endoxifen groups, respectively. However, the corresponding figures for women discontinuing because of an AE related to the IMP were 4, 5, and 11, indicating that the reported AEs might not have been severe, at least not in the 1 mg (Z)-endoxifen group. The most common AE in the group of women discontinuing because of an AE related to the IMP was headache, mostly reported by participants in the 2 mg arm. However, comparing severity of headache at end of study with baseline seen in the answers to the BESS Plus questionnaire, symptoms did not become more severe.

Tamoxifen-induced AEs reported by cancer patients are hard to disentangle from other treatment-related events.^18^ Side effects reported by healthy women on tamoxifen are hot flashes, cold and night sweats, muscle cramps, and vaginal discharge.^19^ We have found no studies reporting endoxifen-specific adverse events, with the exception of patients treated for manic episodes associated with bipolar disorders. Joseph et al. reported headache, nausea, vomiting, and insomnia being the most common side effects in the 330 treated patients included in 3 trials.^20^

Using answers to BESS Plus and KARISMA *Tamoxifen* questionnaires where, in contrast to the AE reporting, baseline and follow-up is compared, we found significantly worsening symptoms of night sweats and hot flashes among participants of the 2 mg (Z)-endoxifen group. In contrast, no significant difference in reported symptoms comparing placebo to the 1 mg (Z)-endoxifen group was seen. Instead, reduced symptoms (diarrhea, vaginal bleeding or spotting, fuzzy vision, breast tenderness, dry mouth, and reduced weight) were reported when the low endoxifen dose was contrasted to placebo. The 2 mg (Z)-endoxifen group showed a significant reduction in muscle stiffness and joint pain.

In the KARISMA *Tamoxifen* trial, women answered a 48-item questionnaire, overlapping with the questions of the *Endoxifen* trial, and used the same Likert scale as in the current study.^19^ The 7 symptoms significantly related to tamoxifen in premenopausal women, when adjusting for baseline values, were hot flashes, cold and night sweats, vaginal discharge, genital itching, muscle cramps, and reduction in breast tenderness. It seems as if there were fewer symptoms significantly related to (Z)-endoxifen compared with tamoxifen using questionnaire data (Table S5).

A weakness of our study is the low participation rate (0.2%), probably explained by the broad invitation of healthy volunteers without prior risk stratification. This risks the generalizability of the particular reported symptom burden. It could be that participants were less likely to report AEs and discontinue than an average group of women. However, this is of lesser importance in a proof-of-concept study where the primary aims were to test if (Z)-endoxifen reduces MBD, to evaluate safety and tolerability, and to determine an optimal dose.

Using MBD change as a proxy for therapy response was chosen for two reasons. It has repeatedly been shown that density decrease is associated with tamoxifen therapy response.^6^^,^^21^ In one of the few prevention studies, it was shown that only participants experiencing a MBD decrease had a reduction in breast cancer incidence.^5^ Second, if using breast cancer incidence as the outcome, several thousand women have to be included, and follow-up has to exceed 5 years. Such a study would be costly, time consuming, and not suitable for a proof-of-concept, dose determination study. However, since MBD change was used as a proxy for therapy response, randomized clinical trials are needed to establish the preventive effect of (Z)-endoxifen.

The reason for including only premenopausal women was that in the previous KARISMA *Tamoxifen* study, we saw an effect on MBD only among younger women^14^—an unexpected finding given that tamoxifen has an established effect on breast cancer recurrence in postmenopausal women^22^ and that density decrease in postmenopausal patients^6^ and healthy individuals^5^ is linked to better outcomes.

We have shown that (Z)-endoxifen at doses of 1 mg and 2 mg lowers MBD to the same extent as 20 mg of tamoxifen, and that the MBD response was seen at plasma concentrations as low as 3 to 4 ng/mL. It seems as if 1 mg (Z)-endoxifen is well tolerated and has a safety profile similar to placebo. The obvious next step is to test if a daily dose 1 mg of (Z)-endoxifen also decreases breast cancer incidence in women at increased risk of breast cancer.

## Supplementary Material

djag087_Supplementary_Data

## Data Availability

The data underlying this article are available on the KARMA Research Platform (karmastudy.org).
