# Supplementary material for: Endoxifen for mammographic density reduction—results from the KARISMA endoxifen trial
Source: J Natl Cancer Inst. 2026 May 4;118(10):1899–906. doi: 10.1093/jnci/djag087 (PMC13650363; doi:10.1093/jnci/djag087)
Supplement: Supplementary file 1 [file djag087_supplementary_data.docx]

**Table S1.** Breast Cancer Prevention Trial (BCPT) Eight Symptom Scale (BESS) Plus 30 – item questionnaire and the KARISMA *Tamoxifen* questions. In all, 36 questions.

We are interested in knowing whether you have had any of the following problems during the past four weeks. Please mark the number which best describes how much each problem bothered you.

|  |  | Not at all | | A little bit | Somewhat | Quite a bit | Very much |
| --- | --- | --- | --- | --- | --- | --- | --- |
| C1 | Difficulty concentrating | | 0 | 1 | 2 | 3 | 4 |
| C2 | Easily distracted | | 0 | 1 | 2 | 3 | 4 |
| C3 | Forgetfulness | | 0 | 1 | 2 | 3 | 4 |
| M1 | Joint pain | | 0 | 1 | 2 | 3 | 4 |
| M2 | Muscle stiffness | | 0 | 1 | 2 | 3 | 4 |
| M3 | General aches and pains | | 0 | 1 | 2 | 3 | 4 |
| V1 | Night sweats | | 0 | 1 | 2 | 3 | 4 |
| V2 | Hot flashes | | 0 | 1 | 2 | 3 | 4 |
| V3 | Cold sweats | | 0 | 1 | 2 | 3 | 4 |
| Ga1 | Vomiting | | 0 | 1 | 2 | 3 | 4 |
| Ga2 | Nausea | | 0 | 1 | 2 | 3 | 4 |
| Ga3 | Diarrhea | | 0 | 1 | 2 | 3 | 4 |
| D1 | Vaginal dryness | | 0 | 1 | 2 | 3 | 4 |
| D2 | Pain with intercourse | | 0 | 1 | 2 | 3 | 4 |
| W1 | Weight gain | | 0 | 1 | 2 | 3 | 4 |
| W2 | Unhappy with the appearance of my body | | 0 | 1 | 2 | 3 | 4 |
| Gy1 | Vaginal discharge | | 0 | 1 | 2 | 3 | 4 |
| Gy2 | Genital itching/irritation | | 0 | 1 | 2 | 3 | 4 |
| Gy3 | Vaginal bleeding or spotting | | 0 | 1 | 2 | 3 | 4 |
| B1 | Difficulty with bladder control (when laughing or crying) | | 0 | 1 | 2 | 3 | 4 |
| B2 | Difficulty with bladder control (at other times) | | 0 | 1 | 2 | 3 | 4 |
| **BESS Plus** | | |  |  |  |  |  |
|  | Headaches | | 0 | 1 | 2 | 3 | 4 |
|  | Blind spots, fuzzy vision | | 0 | 1 | 2 | 3 | 4 |
|  | Breast sensitivity/tenderness | | 0 | 1 | 2 | 3 | 4 |
|  | Weight loss | | 0 | 1 | 2 | 3 | 4 |
|  | Decreased appetite | | 0 | 1 | 2 | 3 | 4 |
|  | Abdominal pain | | 0 | 1 | 2 | 3 | 4 |
|  | Pain or cramps in the legs or feet | | 0 | 1 | 2 | 3 | 4 |
|  | Back pain or problems | | 0 | 1 | 2 | 3 | 4 |
|  | Low energy | | 0 | 1 | 2 | 3 | 4 |
| **KARISMA *Tamoxifen*** | | |  |  |  |  |  |
|  | Rash | | 0 | 1 | 2 | 3 | 4 |
|  | Itching | | 0 | 1 | 2 | 3 | 4 |
|  | Dry mouth | | 0 | 1 | 2 | 3 | 4 |
|  | Fatigue | | 0 | 1 | 2 | 3 | 4 |
|  | Depression | | 0 | 1 | 2 | 3 | 4 |
|  | Lost interest in sex | | 0 | 1 | 2 | 3 | 4 |

**Table S2.** Number of participants, (Z)-endoxifen plasma concentrations at month 1, 3 and 6, in the active (Z)-endoxifen dose arms, in the modified intention to treat population

|  |  | **Randomization arms** | |  |
| --- | --- | --- | --- | --- |
|  | **(Z)-endoxifen 1mg** | | **(Z)-endoxifen 2mg** | |
| **(Z)-endoxifen concentrations, ng/mL** |  | |  | |
|  | **Month 1** | | | |
| Number of participants | 73 | | 75 | |
| Mean (SD) | 5.18 (2.2) | | 10.87 (3.7) | |
| Median | 4.91 | | 10.00 | |
| IQR | 3.78 to 6.12 | | 8.53 to 13.70 | |
| Minimum, maximum | 1.20, 12.90 | | 3.97, 20.10 | |
|  | **Month 3** | | | |
| Number of participants | 69 | | 65 | |
| Mean (SD) | 4.72 (2.2) | | 8.75 (3.7) | |
| Median | 4.48 | | 8.59 | |
| IQR | 3.36 to 5.91 | | 6.34 to 10.70 | |
| Minimum, maximum | 0.42, 13.00 | | 2.47, 19.00 | |
|  | **Month 6** | | | |
| No. | 66 | | 59 | |
| Mean (SD) | 4.75 (1.9) | | 9.69 (2.8) | |
| Median | 4.33 | | 9.79 | |
| IQR | 3.45 to 6.07 | | 7.42 to 11.80 | |
| Minimum, maximum | 0.32, 9.56 | | 2.22, 15.90 | |

**Table S3.** Number of participants and reported adverse events (AEs) considered related to the investigational medicinal product (IMP; assessed as ‘Certain’ or ‘Probable’ by investigator) in the intention to treat population. Symptoms are listed in descending order, stratified per randomization arm. Adverse events reported by more than 1 participant (> 0.4%) shown in the table.

|  | **All** | **Placebo** | **Endoxifen 1 mg** | **Endoxifen 2 mg** |
| --- | --- | --- | --- | --- |
| Number of participants | 240 | 80 | 80 | 80 |
| Number of participants reporting AE’s related to the IMP (%) | 82 (34.3) | 16 (20.0) | 32 (40.0) | 34 (43.0) |
|  | **AE´s related to the IMP**  **No. participants, (%), [No. events]** | | | |
| Irregular menstruation | 29 (12.1) [40] | 3 (3.8) [6] | 13 (16.3) [18] | 13 (16.3) [16] |
| Night sweats | 26 (10.8) [47] | 2 (2.5) [7] | 14 (17.5) [29] | 10 (12.5) [11] |
| Hot flashes | 15 (6.3) [23] | 2 (2.5) [2] | 5 (6.3) [6] | 8 (10.0) [15] |
| Vaginal dryness | 8 (3.3) [8] | 2 (2.5) [2] | 4 (5.0) [4] | 2 (2.5) [2] |
| Cold sweat | 5 (2.1) [5] | 0 (0.0) [0] | 2 (2.5) [2] | 3 (3.8) [3] |
| Vaginal itching | 5 (2.1) [6] | 1 (1.3) [1] | 1 (1.3) [2] | 3 (3.8) [3] |
| Vaginal discharge | 4 (1.7) [9] | 0 (0.0) [0] | 4 (5.0) [9] | 0 (0.0) [0] |
| Dry mouth | 4 (1.7) [4] | 1 (1.3) [1] | 3 (3.8) [3] | 0 (0.0)[0] |
| Difficulty sleeping | 3 (1.3) [4] | 2 (2.5) [2] | 0 (0.0) [0] | 1 (1.3) [2] |
| Headache | 3 (1.3) [4] | 1 (1.3) [1] | 1 (1.3) [2] | 1 (1.3) [1] |
| Joint pain | 3 (1.3) [3] | 1 (1.3) [1] | 2 (2.5) [2] | 0 (0.0) [0] |
| Vaginal bleeding or spotting | 3 (1.3) [3] | 0 (0.0) [0] | 2 (2.5) [2] | 1 (1.3) [1] |
| Dysmenorrhea | 2 (0.8) [5] | 1 (1.3) [2] | 1 (1.3) [3] | 0 (0.0) [0] |
| Depressed mood | 2 (0.8) [2] | 0 (0.0) [0] | 0 (0.0) [0] | 2 (2.5) [2] |
| Excess sweating | 2 (0.8) [2] | 0 (0.0) [0] | 1 (1.3) [1] | 1 (1.3) [1] |
| Muscle cramps | 2 (0.8) [2] | 1 (1.3) [1] | 0 (0.0) [0] | 1 (1.3) [1] |

**Table S4.** Number of participants and reported adverse events (AEs) regardless if the AEs were considered related to the investigational medicinal product (IMP) or not, in the intention to treat population in women *who discontinued* participation. Symptoms are listed in descending order, stratified per randomization arm. Only AEs reported by more than 1 participant (> 0.4%) are shown.

|  | **All** | **Placebo** | **Endoxifen 1 mg** | **Endoxifen 2 mg** |
| --- | --- | --- | --- | --- |
| Number of participants | 240 | 80 | 80 | 80 |
| Number of participants who discontinued | 20 (8.3) | 4 (5.0) | 5 (6.3) | 11 (13.8) |
|  | **AE´s in participants who discontinued**  **No. participants, (%), [No. events]** | | | |
| Headache | 8 (3.3) [10] | 1 (1.3) [1] | 2 (2.5) [3] | 5 (6.3) [6] |
| Night sweats | 4 (1.7) [5] | 0 (0) [0] | 1 (1.3) [1] | 3 (3.8) [4] |
| Joint pain | 4 (1.7) [5] | 1 (1.3) [2] | 2 (2.5) [2] | 1 (1.3) [1] |
| Hot flashes | 3 (1.3) [9] | 0 (0) [0] | 0 (0) [0] | 3 (3.8) [9] |
| Rapid heart rate or palpitations | 3 (1.3) [5] | 0 (0) [0] | 1 (1.3) [2] | 2 (2.5) [3] |
| Constipation | 3 (1.3) [5] | 0 (0) [0] | 2 (2.5) [3] | 1 (1.3) [2] |
| Feeling bloated | 3 (1.3) [3] | 0 (0) [0] | 0 (0) [0] | 3 (3.8) [3] |
| Difficulty sleeping | 2 (0.8) [5] | 0 (0) [0] | 0 (0) [0] | 2 (2.5) [5] |
| Dysmenorrhea | 2 (0.8) [4] | 0 (0) [0] | 1 (1.3) [3] | 1 (1.3) [1] |
| Irregular menstruation | 2 (0.8) [3] | 0 (0) [0] | 1 (1.3) [2] | 1 (1.3) [1] |
| Acne | 2 (0.8) [3] | 0 (0) [0] | 1 (1.3) [2] | 1 (1.3) [1] |
| Back pain | 2 (0.8) [3] | 0 (0) [0] | 0 (0) [0] | 2 (2.5) [3] |
| Nausea | 2 (0.8) [3] | 1 (1.3) [2] | 0 (0) [0] | 1 (1.3) [1] |
| Pain | 2 (0.8) [3] | 0 (0) [0] | 1 (1.3) [1] | 1 (1.3) [2] |
| Vaginal itching | 2 (0.8) [3] | 0 (0) [0] | 1 (1.3) [2] | 1 (1.3) [1] |
| Muscle cramps | 2 (0.8) [2] | 0 (0) [0] | 1 (1.3) [1] | 1 (1.3) [1] |
| Sexual feeling decreased | 2 (0.8) [2] | 0 (0) [0] | 1 (1.3) [1] | 1 (1.3) [1] |
| Tiredness | 2 (0.8) [2] | 1 (1.3) [1] | 0 (0) [0] | 1 (1.3) [1] |
| Upper respiratory tract infections | 2 (0.8) [2] | 0 (0) [0] | 2 (2.5) [2] | 0 (0) [0] |
| Urine incontinence | 2 (0.8) [2] | 1 (1.3) [1] | 0 (0) [0] | 1 (1.3) [1] |
| Weight loss | 2 (0.8) [2] | 1 (1.3) [1] | 0 (0) [0] | 1 (1.3) [1] |

**Table S5.** Number of participants, relative mammographic breast density (MBD) change, mean (Z)-endoxifen concentrations, number of discontinuers and change in side effects in the KARISMA *Tamoxifen* and KARISMA *Endoxifen* trials, in the intention to treat population**s**.

|  | **Endoxifen dose** | | | **Tamoxifen dose** | | |
| --- | --- | --- | --- | --- | --- | --- |
| **Variables** | **0 mg** | **1 mg** | **2 mg** | **5 mg** | **10 mg** | **20 mg** |
| Number of participants | 80 | 80 | 80 | 94 | 95 | 97 |
| Relative MBD change, % | 0.3 | -17.3* | -23.5* | -19.6* | -17.0* | -18.5* |
| Mean endoxifen concentration at end of study, ng/mL | - | 4.8 | 9.7 | 2.4 | 4.5 | 10.1 |
| No. of discontinuers (%) because of an adverse event | 6  (7.5) | 10 (12.5) | 15  (19.0) | 14 (14.9) | 11 (11.5) | 11 (11.3) |
| Mean change in Likert score from baseline to end of treatment^a^ (higher score mean worse) |  |  |  |  |  |  |
| Hot flashes | 0.10 | 0.26 | **0.45*** | **0.62*** | **0.93**** | **0.67*** |
| Night sweats | 0.10 | 0.26 | **0.58**** | **0.60*** | **0.77**** | **0.70**** |
| Cold sweats | 0.04 | 0.12 | 0.10 | 0.12 | **0.34*** | **0.33*** |
| Vaginal discharge | -0.06 | 0.05 | 0.03 | -0.12 | **0.23**** | **0.18**** |
| Genital itching | 0.05 | 0.21 | 0.12 | **0.16*** | **0.15*** | **0.22*** |
| Pain or cramps in legs and feet | 0.14 | 0.20 | 0.17 | **0.22**** | **0.50**** | **0.38**** |
| Sensitive/tender breasts | -0.08 | **-0.18*** | **-0.35*** | **-0.18*** | **-0.13*** | **-0.24*** |

*^a^Seven symptoms identified in premenopausal women in KARISMA Tam when comparing 20 mg tamoxifen to placebo and adjusting for age and baseline value*

** p<0.05 when compared to placebo, ** p<0.01 when compared to placebo*

**Figure S1.** Relative change in mammographic breast density (MBD) from baseline to end of treatment or date of discontinuation, by (Z)-endoxifen concentration in plasma, stratified by randomization arm, in the modified intention to treat population.

**Figure S2.** Spline model describing changes in vasomotor symptom (BESS) as a function of endoxifen concentrations (ng/mL). Randomized (Z)-endoxifen dose in blue vertical dashes. Higher vasomotor score means worse symtoms.
